# Enteral nutrition during bone marrow transplantation in patients with pediatric cancer: a prospective cohort study

**DOI:** 10.1590/S1516-31802012000300005

**Published:** 2012-07-12

**Authors:** Adriana Garófolo

**Affiliations:** I MSc, PhD. Founder and Coordinator of Nutritional Services, Grupo de Apoio ao Adolescente e à Criança com Câncer (GRAACC), Universidade Federal de São Paulo (Unifesp), São Paulo, Brazil.

**Keywords:** Transplants, Enteral nutrition, Neoplasms, Child, Adolescent, Transplantes, Nutrição enteral, Neoplasias, Criança, Adolescente

## Abstract

**CONTEXT AND OBJECTIVE:**

Cancer patients undergoing bone marrow transplantation (BMT) often require nutritional therapy due to treatment toxicities. The aim here was to evaluate the use of tube feeding and its applicability, indications, contraindications and complications in these patients.

**DESIGN AND SETTING:**

Prospective observational study conducted at a public university in São Paulo between January 2002 and August 2007.

**METHODS:**

The patients were followed up daily in the BMT unit by a research dietitian. Tube feeding was indicated when oral supplementation proved to be insufficient, when the patient had severe malnutrition or there was an impediment to use of oral feeding. It was contraindicated in the presence of gastrointestinal toxicity of grade 3 and 4 or other conditions that implied a risk or hindered its use or placement. Complications of tube feeding were divided into minor and major, according to whether they had life-threatening implications.

**RESULTS:**

Forty-two (47.2%) patients had indications for tube feeding: the main reasons were transplantation inadequate food and supplement intake, insufficient intake with malnutrition or weight loss, severe malnutrition or need for oral fasting. Thirty-one (73.8%) received tube feeding: 11 autologous and 20 allogenic patients (P = 0.04). The main contraindications were severe gastrointestinal toxicities and sinusitis. Minor complications from tube feeding were more prevalent in patients with allogenic BMT, but no major complications were observed.

**CONCLUSION:**

Enteral nutrition is a feasible procedure in patients undergoing BMT and should be encouraged. The main difficulty in BMT patients, in relation to tube feeding, is gastrointestinal toxicities.

## RESUMO

**CONTEXTO E OBJETIVO:**

Pacientes com câncer submetidos ao transplante de medula óssea (TMO) frequentemente necessitam de terapia nutricional devido às toxicidades do tratamento. O objetivo foi avaliar uso de sonda enteral, aplicabilidade, indicações, contraindicações e complicações nesses pacientes.

**TIPO DE ESTUDO E LOCAL:**

Estudo observacional prospectivo, conduzido de janeiro de 2002 a agosto de 2007 em uma universidade pública em São Paulo.

**MÉTODOS:**

Os pacientes foram acompanhados diariamente na unidade de TMO por uma nutricionista da pesquisa. Indicações de sonda: suplementação oral insuficiente, desnutrição grave, ou alguma contraindicação para alimentação via oral. A contraindicação da sonda ocorreu na presença de toxicidades gastrintestinais de grau 3 e 4 ou outras condições que implicassem em risco ou impedissem seu uso ou passagem. As complicações da sonda foram divididas em menores e maiores, de acordo com implicações para o risco de vida.

**RESULTADOS:**

Quarenta e dois (47,2%) pacientes tiveram indicações de nutrição por sonda: os principais motivos foram: ingestão alimentar e de suplemento inadequada, ingestão insuficiente com desnutrição ou perda de peso, desnutrição grave ou necessidade de jejum. Trinta e um (73,8%) pacientes usaram sonda: 11 com transplantes autólogos e 20 alogênicos (P = 0,04). As principais contraindicações foram toxicidades gastrintestinais graves e sinusite. Complicações leves com a sonda foram mais prevalentes nos pacientes em TMO alogênico, mas nenhuma complicação grave foi observada.

**CONCLUSÃO:**

O uso de sonda enteral é um procedimento factível em pacientes durante o TMO e deve ser encorajado. A principal dificuldade nesse grupo em relação ao uso de sonda foi a toxicidade gastrintestinal.

## INTRODUCTION

Bone marrow transplantation (BMT) or hematopoietic stem cell transplantation (HSCT) is a recognized therapeutic method for a variety of hematologic malignancies, congenital abnormalities and malignancies. The procedure is used to restore bone marrow function in patients receiving intensive chemotherapy and radiation, through infusion of progenitor cells or stem cells with an ability to multiply and differentiate into all types of mature blood cells: red cells, white cells and platelets.^1^

The transplants may be autologous or allogenic, depending on the origin of the cells. When the cells originate from the patient, the transplant is autologous, but when they are donated by another individual, it is allogenic. If the donor is an identical twin, the transplant is called syngenic.^1^^,^^2^

The complications from BMT can be acute or chronic and depend on the underlying disease and its initial condition before the procedure, the type of transplant, the chemotherapy and the preparatory regimen for radiotherapy. The main post-transplant complications include bleeding, infections, organ failure, graft versus host disease (GVHD), graft failure or rejection, and recurrent disease.^2^

Besides these complications, nutritional status is strongly affected by the process of BMT. The reduced protein intake, for example, may influence immune function during metabolic stress. Thus, studies have demonstrated the importance of adjusting the energy needs to maintain a zero nitrogen balance.^3^^,^^4^^,^^5^

Patients who receive BMT often require nutritional support because of their reduced food intake, which is associated with the toxicities of the conditioning regimen, especially in the gastrointestinal tract.^5^^,^^6^

Patients receiving allogenic BMT undergo a conditioning regimen with high-dose chemotherapy combined with total body irradiation, thereby leading to immunosuppression. The body irradiation is extremely toxic and leads to severe and prolonged mucositis. These patients’ immunity is more highly compromised and they are at greater risk of overall complications than are those who undergo autologous BMT. Therefore, it is believed that patients who undergo allogenic BMT are also at greater risk of nutritional and metabolic complications and therefore need specialized nutritional support.^7^ Total parenteral nutrition (TPN) has been the method most commonly used to provide nutrients during BMT. However, circumstances such as risk of infection and lipid and glucose metabolism disorders may limit its use in these patients.^8^

## OBJECTIVE

Since it is known that patients undergoing BMT often require nutritional support due to treatment toxicities, the objective of the present study was to evaluate the use of enteral nutrition through tube feeding and its applicability, indications, contraindications and complications in patients undergoing BMT, with comparisons between autologous and allogenic transplants.

## METHODS

This was a prospective observational study carried out from January 2002 to August 2007, among patients admitted to undergo autologous or allogenic BMT procedures. The study was conducted in accordance with a follow-up protocol from the BMT research unit at a pediatric oncology institute in a public university in São Paulo.

The study included all patients diagnosed with cancer for whom BMT was indicated, and the study excluded those whose family or legal guardian (and the patient, if over 18 years) had not agreed to sign the consent form, thereby refusing to participate. Patients who had been referred for transplantation but were not suffering from cancer were also excluded.

The patients were followed up daily by a research dietitian, using a standardized report form schedule that had been developed to gather data on food intake; gastrointestinal signs and symptoms; oral supplements; administration of and indications and contraindications for enteral and parenteral nutritional support; the antineoplastic treatment protocol; and other treatments. To estimate the total daily energy requirement, we used the World Health Organization (WHO) or Harris Benedict equation, according to the age group.^9^^,^^10^^,^^11^ A calculation of baseline energy expenditure (EE) plus a factor of 1.2 (illness) was applied in order to obtain the total EE (TEE).

 Weight-for-height (W/H) Z-scores from < -1.0 to -2.0 among children^12^ and body mass index (BMI) from greater than or equal to the fifth percentile to greater than the fifteenth percentile among adolescents^13^ were considered to represent mild malnutrition; and W/H Z-scores < -2.0 in children and less than the fifth percentile of BMI in adolescents were considered severe.^14^ In young adults, the WHO cutoff values^15^ were applied: < 18.5 for mild and < 17 for severe malnutrition.

### Nutritional management

Indications for nutritional support were made in accordance with the routine protocol of the transplantation unit. They were applied by the nutrition team and consisted of indications of nutritional supplementation when the food intake was insufficient (less than 70-80% of energy needs) for three to five days.

Enteral tube feeding was indicated when: oral food and supplementation proved to be inadequate (less than 50% of energy needs); the patient had insufficient food and supplement intake, with malnutrition or weight loss; the patient had severe malnutrition or he or she had an impediment to use of oral feeding. After the nutrition team had made an indication for tube use, the patient underwent a clinical evaluation by the oncologist and the nursing staff, in order to implement this procedure.

Placement or use of the tube was contraindicated by the medical staff if the patient presented gastrointestinal toxicity of grade 3 or 4, or other medical complications that resulted in risk or could impede its use or placement. Parenteral nutrition was therefore administered when previous procedures had not been successful or were contraindicated.

The complications of tube feeding were divided into minor and major, according to whether they had life-threatening implications. Minor complications, referred to as non-life-threatening, comprised categories such as problems with the tube (inadvertent tube dislodgement or tube clogging), intensification of episodes of vomiting or diarrhea, colic or abdominal pain and infection in the oral cavity. Major complications were those that were systemic and life-threatening, and included malpositioned feeding tubes involving inadvertent placement into the respiratory tract, bleeding or injury in the oral cavity or gastrointestinal tract, perforation of the esophagus or stomach, disrupted breathing (obstruction of nasal breathing), sinusitis, epistaxis or aspiration pneumonia.^16^

### Statistical analysis

The data were registered and analyzed using the NCSS/PASS statistical software.^17^ Differences between groups were investigated using the Mann-Whitney test for continuous variables. For categorical variables, the chi-square test was applied.

The sample size was calculated considering an estimated rate of 30% for the malnutrition outcome in the autologous group and 60% in the allogenic group, a significance level of 5% (chance of type 1 error or alpha = 0.05) and a power of 80% (chance of type 2 error or beta = 0.2).

### Ethics committee

The nutritional support protocol from which this analysis was derived was approved by the Institutional Review Board/Ethics Committee of Universidade Federal de São Paulo - Escola Paulista de Medicina (Unifesp-EPM), under protocol number 0132/06. Informed consent was obtained from the parents, guardians or patients, after the study protocol had been explained to them.

## RESULTS

Eighty-nine patients out of the 101 followed up during the study period fulfilled the inclusion criteria: 45 autologous transplant cases (50.6%) and 44 allogenic transplant cases (49.5%). Of these, forty-two (47.2%) had an indication for tube feeding: 18 autologous and 24 allogenic cases (Table 1). The patients’ demographic characteristics are shown in Table 2.

**Table 1.** Cancer diagnoses according to the type of bone marrow transplant (BMT)

BMT type	Diagnoses	Number of patients	Percentage
Autologous		n = 18	
	Acute myeloid leukemia	1	5.6
	Hodgkin’s lymphoma	8	44.4
	Non-Hodgkin’s lymphoma	4	22.2
	Germ cell tumor	1	5.6
	Neuroblastoma	3	16.7
	Ewing’s sarcoma	1	11.1
Allogenic		n = 24	
	Acute lymphoblastic leukemia	15	62.5
	Chronic myelomonocytic leukemia	1	4.2
	Acute myeloid leukemia	5	20.8
	Non-Hodgkin’s lymphoma	1	4.2
	Biphenotypic leukemia	1	4.2
	Ewing’s sarcoma	1	4.2
Total		42	100

**Table 2.** Demographic characteristics of the patients (n = 31)

Transplant	Male	Female	Children^*^	Adolescents^†^	Adults^‡^
Autologous (n = 11)	6 (55%)	5 (45%)	3 (27%)	6 (55%)	2 (18%)
Allogenic (n = 20)	13 (65%)	7 (35%)	12 (60%)	6 (30%)	2 (10%)
Total	19	12	15	12	4

^*^Children: 0-9 years; ^†^Adolescents: 10-18 years; ^‡^Adults: over 18 years.

The mean of length of hospital stay was 30.4 days for autologous and 37.2 days for allogenic transplant patients (P = 0.06).

Among the patients who underwent autologous transplantation, the main reasons for the tube indication were inadequate oral food and supplement intake in eleven (61%), insufficient supplement and food intake with malnutrition or weight loss in five (28%) and severe malnutrition in two (11%). Among the patients who underwent allogenic BMT, the reasons were: inadequate oral food and supplement intake in 17 (70.8%), insufficient oral food and supplement intake with malnutrition or weight loss in four (16.7%) and need for oral fasting in three (12.5%). Comparing the autologous and allogenic groups, there was no difference in the numbers of patients with inadequate food intake or with malnutrition and weight loss. Table 3 shows the nutritional status of the group.

**Table 3.** Nutritional diagnosis of the patients before bone marrow transplant (at admission) and afterwards (at discharge) (n = 31)^*^

Bone marrow transplantation type	Nutritional status	At admission	Percentage	At discharge	Percentage
Autologous (n = 11)	Severe malnutrition	2	18	1	9
	Mild malnutrition	2	18	3	27.3
	Normal weight	7	63.6	6	54.5
	Overweight/obese	0	0	0	0
Allogenic (n = 20)	Severe malnutrition	2	10	1	5
	Mild malnutrition	0	0	0	0
	Normal weight	12	60	12	60
	Overweight/obese	6	30	4	20
Total (n = 31)	Severe malnutrition	4	12.9	2	6.5
	Mild malnutrition	2	6.5	3	9.7
	Normal weight	19	61.3	18	58
	Overweight/obese	6	19.4	4	12.9

^*^Four patients were lost at discharge: one autologous case with normal weight at admission and three allogenic cases (one obese individual and two with normal weight at admission).

In the light of these indications, 31 (73.8%) received tube feeding and 11 (26.2%) did not. Out of the 31 patients with the tube, 11 were autologous and 20 were allogenic cases (P = 0.04).

Among the autologous cases, the reasons associated with not using tube feeding were non-acceptance by the patient or family in three cases, severe gastrointestinal toxicities in two, high risk of intestinal perforation in one and sinusitis in one. Among the allogenic cases, the reasons were the presence of significant sinusitis in two cases, gastrointestinal toxicity in one and high-output colostomy in one.

Twelve patients (38.7%) received tube placement on day zero of BMT (day of infusion of bone marrow cells) and 19 (61.3%) after that day, ranging from one to 20 days later. The average duration of tube use was 14 days for allogenic cases and 20 days for autologous cases (P = 0.08).

**Graph 1.**
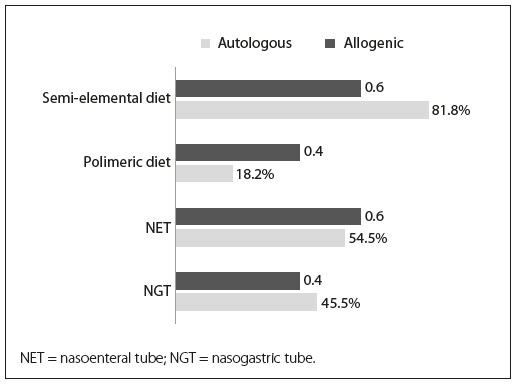
Description of feeding tube placement and type of diet according to the transplant.

A semi-elemental diet was required at some time during the treatment in 21/31 patients (67.7%): 81.8% of the autologous cases and 60% of the allogenic cases (difference not significant). The descriptions of the type of diet and tube position according to group are shown in Graph 1. No analysis was performed on the infusion of enteral diet.

Twenty patients only used the feeding tube as an addition to the oral route; the sum of these two routes contributed 79.6% of TEE. The average adequacy of energy intake only with tube feeding was 51% for the whole group: 56% versus 38% for the autologous and allogenic cases, respectively (P = 0.08); 39.7% of the patients used a nasogastric tube feeding (NGT) and 46% used a nasoenteral tube (NET).

There were no major complications from tube feeding, but some minor complications were observed in 17 patients (54.8%): intensification of the episodes of vomiting or diarrhea with progression of the volume of diet in five (16.1%), tube dislodgement in six (19.4%), fungal infection in the oral cavity in three (9.7%) and problems with the tube (tube clogging) in two (6.5%).

Comparing the complications between the autologous and allogenic cases (36% versus 60%; not significant), intensification of the episodes of vomiting or diarrhea was found in one (9.1%) versus four (20%), respectively. The three patients with fungal infection in the oral cavity belonged to the allogenic group. Tube clogging and tube dislodgement were found in 9% versus 5% and 18.2% versus 20% in the autologous and allogenic cases, respectively (not significant).

## DISCUSSION

Currently, hematopoietic stem cell transplantation may be an alternative to conventional treatment that can be indicated for treating various malignant childhood tumors. According to a recent consensus, the main indications in pediatric oncology patients may be some leukemias and lymphomas and some solid tumors such as neuroblastoma, some central nervous system tumors, Wilms’ tumor, germ cell tumors and some bone tumors such as Ewing’s sarcoma.^18^

Patients undergoing this procedure are at greater nutritional risk because of the aggressiveness of the therapy. Gastrointestinal toxicities such as mucositis, nausea and vomiting are observed in all patients undergoing BMT.^19^

Parenteral nutrition is a method of nutritional therapy that is widely used and recommended as a choice for patients who undergo BMT, mainly because of the results of an earlier study that demonstrated that its prophylactic use had a positive impact on patient survival after three years of follow-up.^20^ However, the use of total parenteral nutrition (TPN) is also associated with an increased risk of complications, especially infectious and metabolic diseases, for which the greatest risk may be among patients with severe immunosuppression, such as BMT patients. Therefore, routine use of TPN is not warranted, unless toxicity or serious complications of the gastrointestinal tract, preclude the full use of enteral nutrition.^21^^,^^22^^,^^23^^,^^24^

Therefore, nutritional therapy has been widely recommended for pediatric patients undergoing BMT, and enteral nutrition through a feeding tube is preferred over TPN in the absence of severe mucositis.^25^ In the present study, severe gastrointestinal toxicities and sinusitis were the main problems that contraindicated placement of tube feeding among the allogenic and autologous BMT patients. However, this group of patients who experienced such problems was receiving the only alternative to parenteral nutrition for support.

Several research groups have advocated the use of percutaneous endoscopic gastrostomy (PEG) for cancer patients, especially when nutritional therapy is required for a prolonged period.^25^^,^^26^^,^^27^ Although this method has not been tested in BMT patients, it may be useful and feasible if used early, before lesions occur in the oral mucosa and gastrointestinal tract thereby preventing tube placement in situations of aplasia with severe thrombocytopenia. Even though all the procedures for tube placement were followed, in accordance with the indications and contraindications, it was observed that more than 60% of the patients underwent tube placement after infusion of bone marrow cells, i.e. when the blood cell count was already very low and the lesions in the gastrointestinal mucosa were starting to emerge. Delay in indication of nutritional support may hamper the use of tube feeding, and predispose towards increased risk of complications. Therefore, early indication of tube feeding could benefit a greater number of patients, thereby reducing the need for TPN, or at least reducing its length of use.

On the other hand, some groups of experts are resistant to using tube feeding in BMT patients, believing that the procedure has a high risk due to gastrointestinal toxicities and the low platelet and leukocyte counts that occur in these individuals.^4^ Although usually considered to be a harmless procedure, blind placement of tube feeding may result in serious and even lethal complications. Given the widespread use of tube feeding in patients of all ages, even a small percentage of such problems may affect a significant number of people.^16^ In this study, tube feeding was used without major complications, thereby contributing 51% of TEE and demonstrating that the method was feasible in this population of cancer patients.

Enteral nutrition has been extensively recommended for adults and infants during cancer treatment.^28^^,^^29^^,^^30^ Several studies have found it to be feasible in patients with cancer undergoing BMT, with favorable evolution of nutritional status achieved by means of feeding tubes.^31^^,^^32^^,^^33^ Langdana et al. found that nutritional therapy through an aggressive program of enteral nutrition using feeding tubes was possible in a pediatric population that underwent BMT, including patients who received conditioning with total body irradiation. They noted that this method contributed between 33% and 48% of energy requirements per kilogram of weight during the program.^34^

The main indications for use of tube feeding found in the present study were low food intake and malnutrition. Such conditions are common in cancer patients who undergo BMT. Therefore, nutritional support is crucial during this period in order to ensure a more favorable response to treatment, thereby improving the chances of cure and survival.

Patients receiving allogenic transplants underwent conditioning regimens that were more aggressive, usually consisting of a combination of extremely high doses of two or more chemotherapy regimens, often in combination with total body radiation. Such therapy is extremely toxic, with serious consequences for the integrity of the gastrointestinal tract, and various other toxic effects. Comparing the autologous and allogenic groups, no difference was found in relation to the causes of indication, i.e. the numbers of patients with inadequate food intake or with malnutrition or weight loss were not different. The average percentage of patients with adequate energy intake was 56% in autologous and 38% in allogenic cases, which demonstrated a borderline difference. This difficulty may be associated with the type of transplant, thus showing that patients who underwent allogenic BMT were at higher nutritional risk.

In this study, no differences in the reasons that contraindicate use or placement of tube feeding could be perceived between the types of BMT. However, with the exception of the refusal of patients or relatives to undergo this procedure, the main reasons that did not allow the use of tube feeding in both groups were severe gastrointestinal problems and the presence of sinusitis.

Patients undergoing BMT procedures are at increased risk of organ toxicity due to the aggressiveness of the therapeutic implants. Gastrointestinal toxicities such as mucositis, nausea and vomiting are expected in patients undergoing BMT.^19^ Several patients in this study had tube placement contraindicated for reasons relating to BMT complications, which frequently occur over the two weeks following infusion of marrow cells. This probably contributed towards the lower success of enteral nutrition. Even transfusion of platelets in the presence of severe thrombocytopenia or administration of medications to control vomiting, in order to reduce the risk of tube feeding and improve the therapy, did not ensure full infusion volume of the diet planned to meet the requirements. This probably contributed towards the results in terms of energy supply, thereby reducing the efficacy of the therapy.

Thus, this study highlights the importance of developing clinical trials using tube feeding and PEG at the beginning of the BMT treatment. This could provide nutritional support with less risk of infections and less use of TPN or its use for a shorter period.

Moreover, early placement of percutaneous endoscopic gastrostomy (PEG) would eliminate the risks associated with the procedure for placing tubes in situations of marrow aplasia and severe toxicities of the gastrointestinal tract, as well as contraindications for placement of the tube, for the same reason. Therefore, this approach could ensure a more adequate supply of enteral diet throughout the period of transplantation, without loss of the tube accidentally or through vomiting, with increased diet tolerance, decreased risk and a positive impact on nutritional status and consequently on the prognosis.

Because of the frequency of gastrointestinal toxicities in these patients, especially mucositis and enteritis, changes in the formulation of enteral feeding may be necessary in order to facilitate tolerance. In many cases, diets that are easier to digest and absorb are used. In this study, we observed that a semi-elemental diet was required at some time during the treatment in 81% of the autologous and 60% of the allogenic BMT patients.

Bone marrow toxicity, which leads the patient to bone marrow failure, is one of the biggest challenges faced by the team, because of the high risk of death in this group. There is evidence that allogenic transplantation contributes more to the risk factors for overall complications, mainly due to increased inflammatory response, with higher synthesis of pro-inflammatory cytokines during this phase. This condition seems to be further aggravated in allogenic unrelated transplantation, which was not included in this study because, at that time, the institution had not yet performed this type of BMT.^8^^,^^35^

Although no significant differences between autologous and allogenic cases were observed in this study, regarding the presence of minor complications from use of tube feeding, the patients who underwent allogenic BMT had a greater percentage of these complications, which were mainly gastrointestinal and infectious diseases, thus corroborating the data in the literature.

The results also showed that patients who underwent allogenic BMT had more difficulty in achieving energy supply with nutritional support. This could be correlated with the greater number and severity of complications in this group, thereby also indicating higher nutritional risk and a worse clinical outcome.

Finally, enteral nutrition through the tube was seen to be a feasible procedure in patients undergoing BMT. This procedure should be encouraged, in keeping with the recommendations for care in relation to gastrointestinal toxicities, mucosal lesions, risk of bleeding and other conditions that lead patients to the risk of acquiring infections.

Moreover, early indication, correct choice of enteral feeding, specific care during tube placement procedures, attention to production and administration of diet and appropriate use of medications can optimize the tube feeding method. Nevertheless, in the event of severe gastrointestinal toxicities, use of TPN should be considered when the patient requires nutritional support and tube feeding is temporarily contraindicated.

Hence, there is a need for controlled trials to assess the applicability and benefits of nutritional support, in particular regarding the use of tube feeding and PEG, and especially through investigating early indication.

## CONCLUSION

Enteral nutrition is a feasible procedure in patients undergoing BMT and should be encouraged. The main difficulty faced by BMT patients in using tube feeding is gastrointestinal toxicities. It is important to conduct controlled clinical trials to assess the applicability and benefits of nutritional support, and in particular the applicability of tube feeding and PEG, with investigations on early indication.
